# The association between vertical laminar fracture and recurrent kyphosis after implant removal of Thoracolumbar burst fracture: a retrospective study

**DOI:** 10.1186/s12891-023-06139-w

**Published:** 2023-01-21

**Authors:** Hualin Li, Qi Chen, Jiasen Hu, Jiapei Yu, Jianwei Xiang, Kaiyu Li, Junjie Weng, Naifeng Tian

**Affiliations:** 1 The Second Affiliated Hospital and Yuying Childrens Hospital of Wenzhou Medical University, Zhejiang Province, China; 2 The Third People’s Hospttal Of Qingdao, Qingdao Province, China

**Keywords:** Burst fracture, Laminar fracture, Recurrent kyphosis, Thoracolumbar fracture

## Abstract

**Background:**

Surgeons often encounter recurrent kyphosis of Cobb angle following thoracolumbar burst fracture surgery. Some factors affecting postoperative correction loss have been studied in previous studies, but few have examined the relationship between laminar fractures and postoperative loss of correction.

**Methods:**

The clinical data of 86 patients with thoracolumbar burst fracture who met the inclusion criteria and were admitted to our Department of Spine Surgery between 2013 and 2020 was retrospectively analyzed. To examine the association between laminar fracturs and postoperative correction loss, demographic and radiographic characteristics of the two groups were analyzed.

**Results:**

The presence or absence of laminar fractures was statistically different between the two groups (*P* < 0.05). Binary logistic regression analysis showed that laminar fractures and preoperative Cobb were statistically significant in the two groups. There were statistically significant differences in the degree of injury of laminar fractures in the coronal plane between the two groups (*P* < 0.05).

**Conclusion:**

This study investigated that the presence or absence of laminar fractures and preoperative Cobb contribute to loss of correction after thoracolumbar burst fracture surgery. There was a statistically significant difference between full-length and partial-length laminar fractures on the loss of postoperative correction of thoracolumbar burst fractures with laminar fractures.

## Background

In the thoracolumbar spine segment, the “comparatively inactive” thoracic vertebrae meet the “comparatively active” lumbar vertebrae, including T11, T12, L1, and L2, which is an area of stress concentration. It is reported that nearly 90% of vertebral fractures occur in this are [1–3], among which burst fractures account for 30-60% of thoracolumbar fractures [4, 5]. High-energy injuries, such as falling from a height, road traffic accidents, and being struck by heavy objects, are the most common injuries. The spine is subjected to tremendous longitudinal compression in the injury process, causing the vertebral body to burst into many bone fragments. This compression force is transmitted from the vertebral body along the pedicle to the lamina, eventually leading to the lamina fissure. The trauma sustained by fractures with lamina fissures usually results in more severe injury than fractures without lamina fissures.

Skiak E et al. [6] classified the laminar fractures into complete and incomplete. Laminar fractures that involve only a single layer of the bone cortex or occur only on one side of the proximal spinal canal are called laminar fractures. Shi et al. [7] demonstrated the association.

of the presence and its types of lamina fractures with posterior dural tear and neurological deficits in traumatic thoracic and lumbar burst fractures. Accordingly, most current studies are based on this classification, which is solely based on the CT axial level laminar fracture morphology. A limited number of studies have been reported on the morphology of laminar fractures seen in other CT planes. Xu et al. [8] examined laminar fracture morphology in multiple CT planes and found vertical laminar fracture to be associated with neurological deficits and TLICS scores. The study by Mohamed M. Aly et al. [9] showd VLF to be associated with A4 fractures and radiographic parameters such as vertebral height loss and canal compromise percentage. There is no widely recognized precise prediction system of whether recurrent Cobb angle will occur after thoracolumbar burst fracture due to the simultaneous presence of multiple risk factors. The laminar fracture is seldom discussed as an independent risk factor for postoperative loss of correction. The study objective is to examine the association of vertical laminar fracture and its morphological variations with progressive kyphosis after instrumentation removal.

## Methods

### Inclusion/exclusion criteria

The following inclusion criteria were used in this study: (1) Presence of a clear history of trauma and time to injury less than or equal to one week; (2) vertebral burst fracture supported by radiographic examination; (3) ASIA grade E; (4) injury of single segments of thoracolumbar junction fractures (T11-L2); (5) no history of thoracolumbar surgery; (6) non-pathological or osteoporotic fractures;(7) no PLC injuries.

### Imaging evaluation

**Fig. 1:**
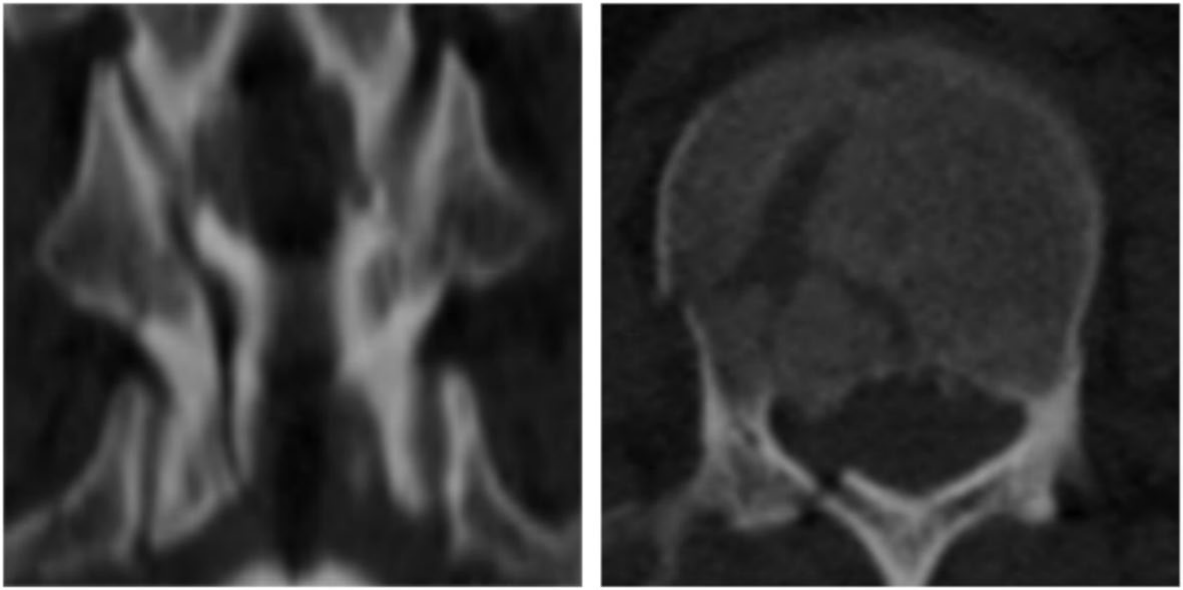
Complete laminar fractures. CT axial and coronal planes show the fracture line through all lamina

**Fig. 2:**
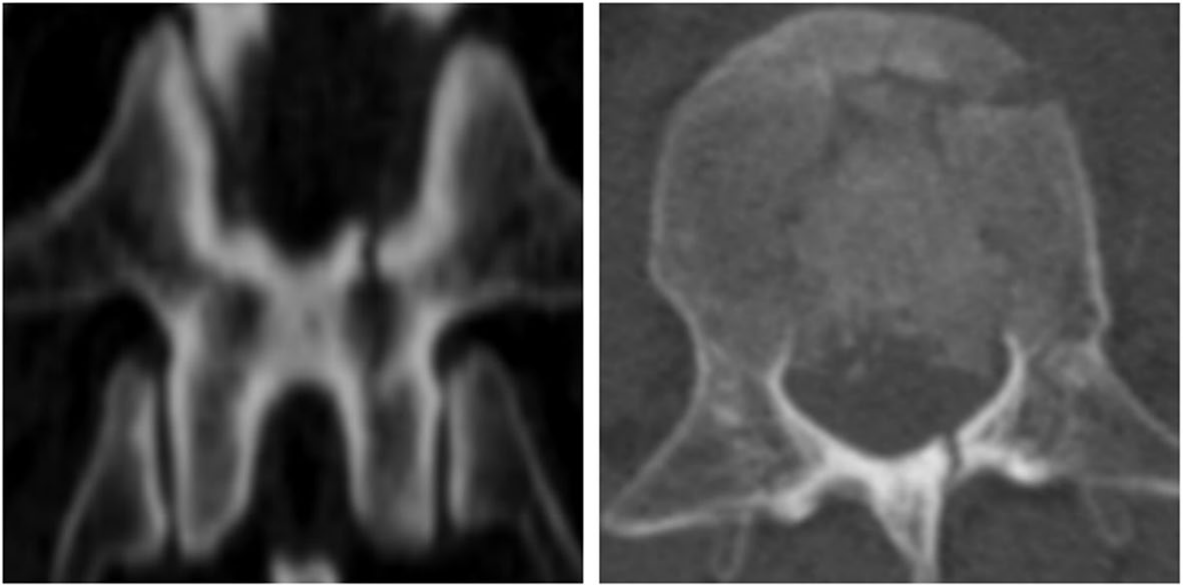
Partial and full-length laminar fractures. Reconstructed coronal computed tomographic scan revealing full-length laminar fracture. The axial plane shows complete or incomplete laminar fracture

**Fig. 3:**
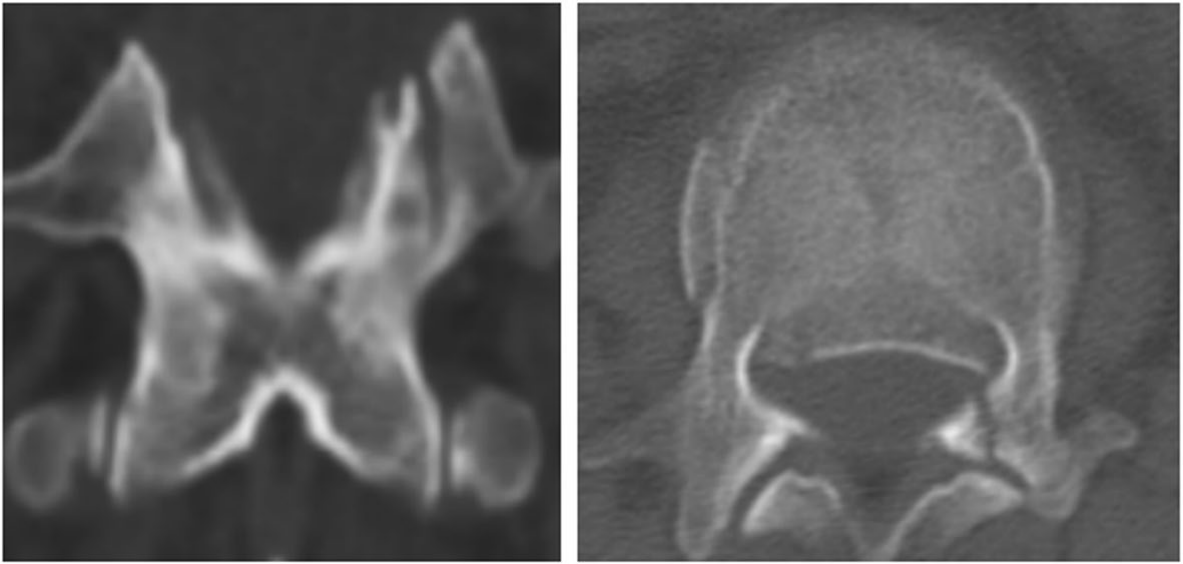
Partial and part-length laminar fractures. Reconstructed coronal computed tomographic scan revealing part-length laminar fracture. The axial plane shows complete or incomplete laminar fracture

**Fig. 4:**
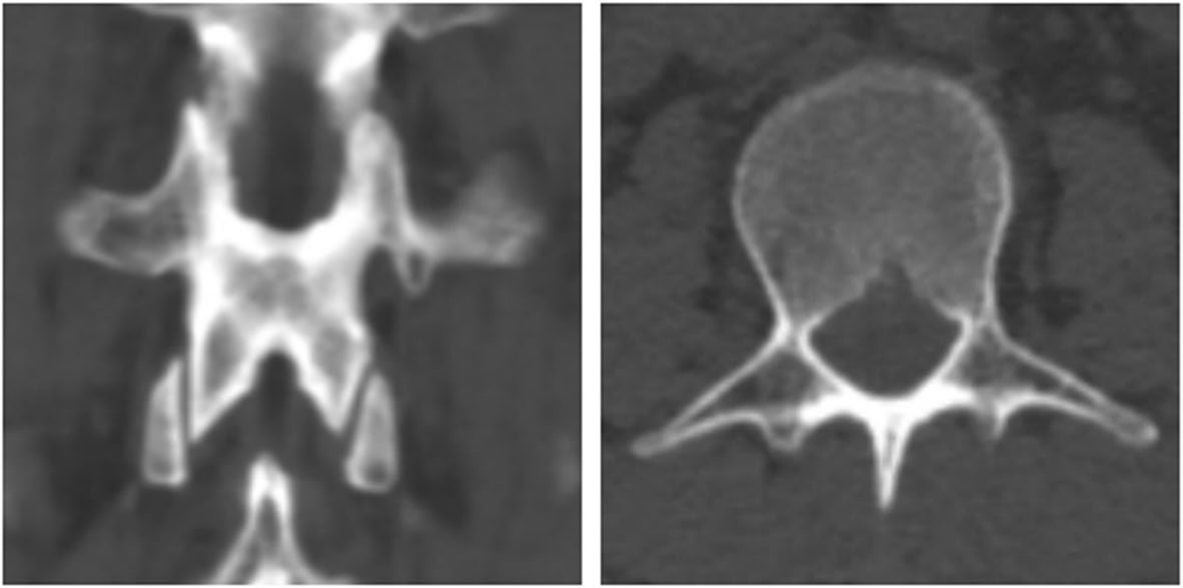
No laminar fractures. CT axial and coronal planes show no laminar fractures

**Fig. 5:**
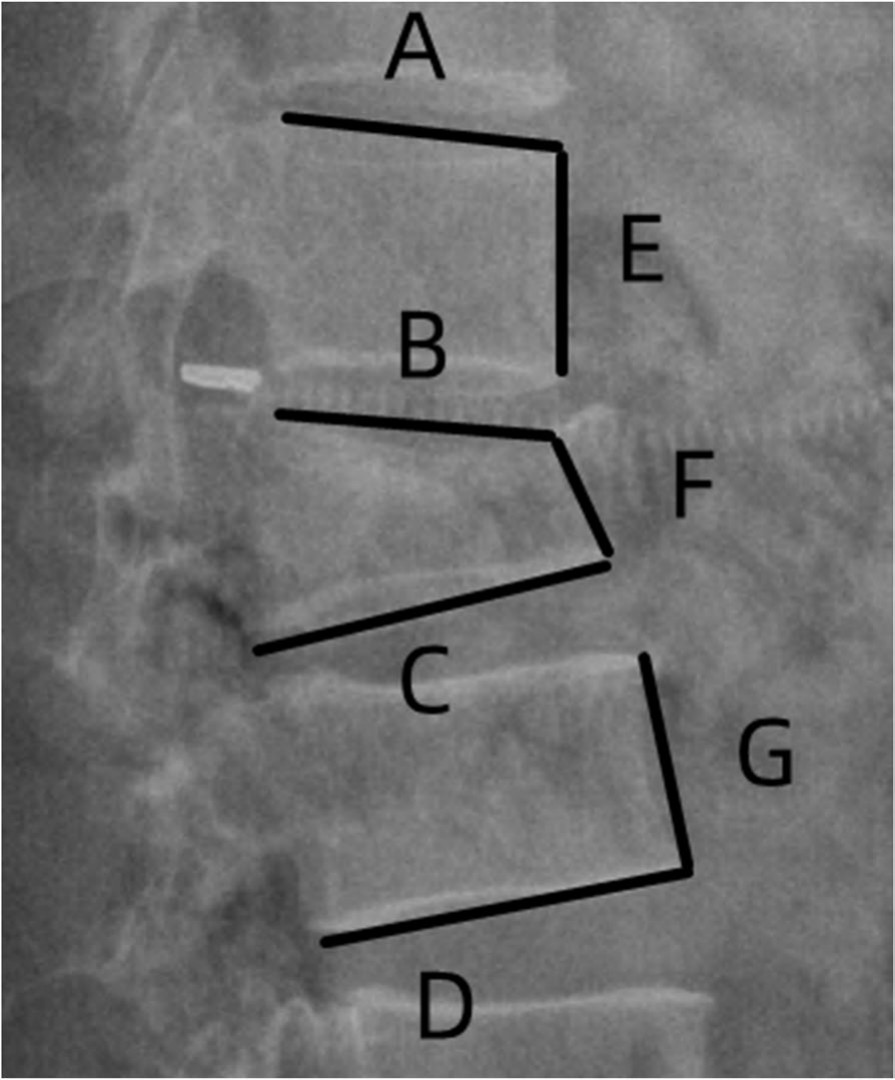
The angle between the two lines of **A** and **D** was the kyphotic Cobb angle. The angle between the lines of **B** and **C** was vertebral wedge angle (VWA). The length of segment E was the height of the anterior edge of the vertebral body superior to the injury, the length of segment F was the height of the anterior edge of the injured vertebral body, and the length of segment G was the height of the anterior edge of the vertebral body inferior to the injury. AVH% = 2F/(E + G)*100

In this study, 2D CT reconstructions were performed for all patients preoperatively. The severity of the injury was assessed preoperatively using Asia grade [10], LSC score [11], TLICS score [12], and the treatment regimen was determined. After reviewing the evaluation results, all patients must undergo a short segmental pedicle screw fixation. We used the categories“Complete laminar fractures”, “Partial laminar fractures”, and “No laminar fractures” to describe the morphology of the lamina in CT. Furthermore, we classified these reconstructions into the following four categories: Complete laminar fractures (Fig. 1), partial and full-length laminar fractures (Fig. 2), partial and partial-length laminar fractures (Fig. 3), and no laminar fractures (Fig. 4). The kyphotic Cobb angle was obtained by measuring the angle between the upper endplate horizontal extension line of the adjacent superior vertebral body of the injured vertebra and the lower endplate horizontal extension line of the adjacent inferior vertebral body of the injured vertebra [13]. Vertebral wedge angle (VWA) was obtained by measuring the angle of the horizontal extension line at the upper and lower endplates of the injured vertebrae [13]. AVH% of injured vertebrae = (the height of the anterior edge of injured vertebrae/the average height of the anterior edge of the upper and lower vertebrae adjacent to the injured vertebrae) * 100%(Fig. 5). All imaging data in this study were obtained through the picture archiving and communication system (PACS; INFINITT PACS; Infinitt, Seoul, Korea) measurements. Two independent surgeons made separate measurements without specifying the applications of the measured data, but all discordant opinions were resolved by the average measurement.

### Study setting

**Fig. 6:**
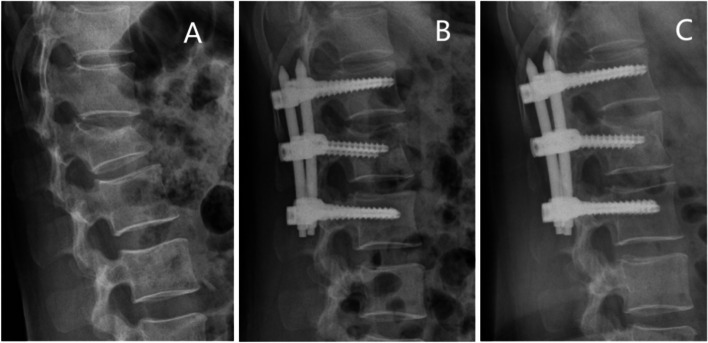
Non-recurrent kyphosis group. Typical case: female, 49 years old, burst fracture of L2 vertebral body. Panel **A** shows a preoperative lateral radiograph of the lumbar spine. Panel **B** is a postoperative lateral radiograph of the lumbar spine showing a kyphotic Cobb angle of 1.05°.Panel **C** is a lateral radiograph of the lumbar spine before internal fixation was removed, showing a kyphotic Cobb angle of 1.67°.This suggested that the kyphotic Cobb angle was lost less than 5°before the last follow-up visit or internal fixation was removed compared with the immediate postoperative kyphotic Cobb angle loss

**Fig. 7:**
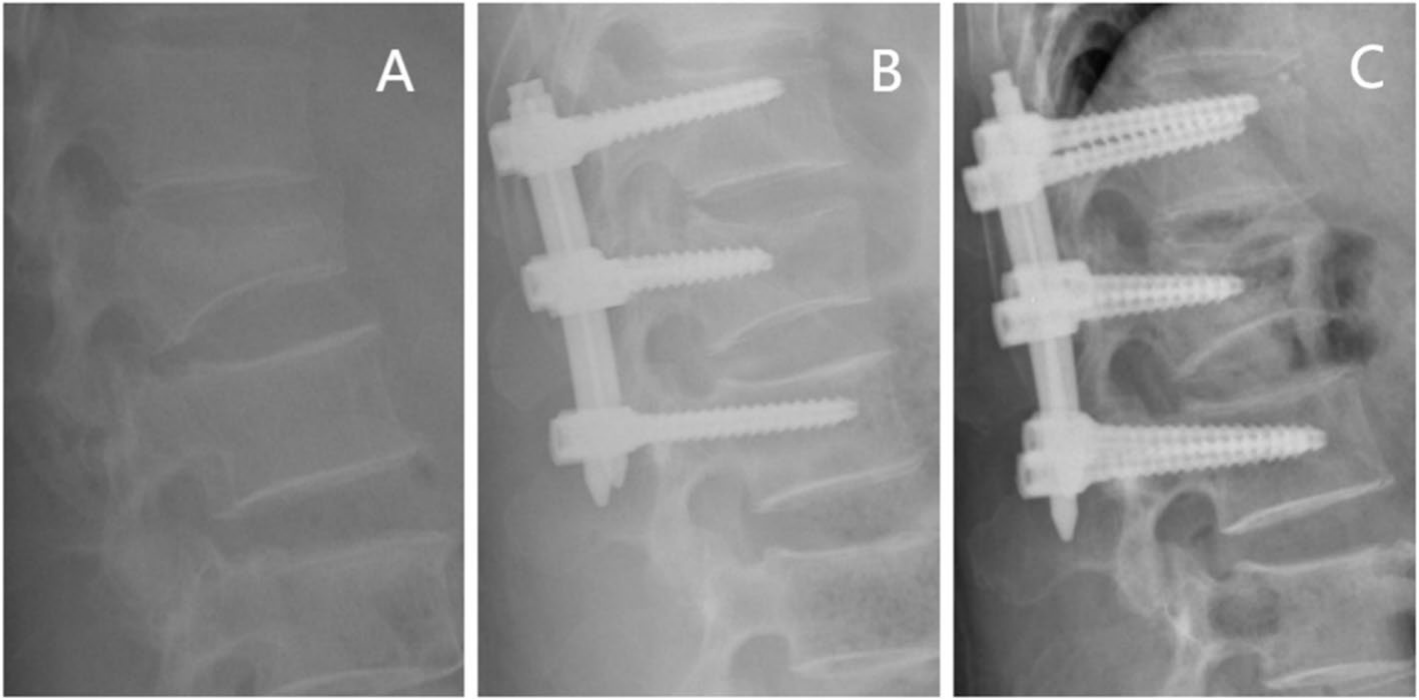
Recurrent kyphosis group. Typical case: male, 51 years old, burst fracture of L1 vertebral body. Panel **A** shows a preoperative lateral radiograph of the lumbar spine. Panel **B** is a postoperative lateral radiograph of the lumbar spine showing a kyphotic Cobb angle of 4.72°. Panel **C** is a lateral radiograph of the lumbar spine before internal fixation was removed, showing a kyphotic Cobb angle of 16.19°.This suggested that the kyphotic Cobb angle was lost more than 5°before the last follow-up visit or internal fixation was removed compared with the immediate postoperative kyphotic Cobb angle loss

The patients were prohibited from heavy physical labor and strenuous exercise for six months postoperatively. After Intraprocedural fixation, radiographic data were collected before or at the last follow-up visit. In the immediate postoperative period, the Cobb angle in the lateral radiograph of these patients was compared to that of the lateral radiograph. The patients were divided into two groups according to whether the angle of loss was greater than 5 degrees [14]: A group (recurrent kyphosis) and B group (Non-recurrent kyphosis), to reveal the independent risk factors for loss of postoperative correction. Two typical cases are shown in Figs. 6 and 7.

### Surgical procedures

Before the surgery was performed, the fracture segment was re-identified using a C-arm machine; routine preoperative disinfectant drapes were placed. The incision along the dorsal midline was approximately 10 cm long. The blunt tissue was separated layer by layer using the intervertebral space approach. The fractured vertebral body and the pedicle screw pinning points of the adjacent upper and lower vertebral bodies were exposed. To determine the trajectory of the screw, an opener was used to pass through the bone cortex, the nail canal was perforated, and the nail canal’s four walls were probed with a probe to ensure that all pedicles had not been breached. The screw trajectory was again determined fluoroscopically. To select the appropriate pedicle screw based on the patient’s pedicle size, four pedicle screws manufactured by the same company (length 40-50 mm; 6.0-6.5 mm in diameter) were placed in the pedicle screw canals of the adjacent upper and lower vertebral bodies of the injured vertebrae at appropriate lengths, and two pedicle screws (length is 30-35 mm; 5.5-6.0 mm in diameter) manufactured by the same company were placed into the pedicle screw canals of the injured vertebrae at appropriate lengths. C-arm fluoroscopy demonstrated satisfactory results for the position and orientation of the pedicle screws and the damaged vertebral body. Next, a horizontal link was mounted with wound irrigation with a copious amount of normal saline. There was no evidence of active bleeding, and it was covered with gel foam. After the bandaging was placed, a negative pressure drainage tube was placed, the incision was closed in layers, and the bandaging was subsequently fixed with a sterile dressing. After these steps were followed, the surgical operation was deemed complete [15–17].

### Statistical analysis

All data were analyzed with SPSS version 26.0 (SPSS, Chicago, Illinois, USA). The dependent variables are gender,location,aetiology,laminar fractures,age,follow up time,pre-AVH%,pre-cobb angle and pre-VWA.Continuous variables were compared using the t-test. Pearson’s chi-squared test and continuity corrected chi-square test compared categorical data, with *P* < 0.05 as the testing criterion. Binary logistic regression was used to determine whether the presence or absence of laminar fractures was associated with postoperative loss of correction. The independent variable for logistic regression is Kyphosis> 5 degrees on the latest follow-up. Before binary logistic regression analysis, we included variables that were significantly different (*P* < 0.05) between the groups in the final binary logistic regression analysis, and variables were removed if they failed to meet the criteria of *P* < 0.05, and the results were reported as 95% confidence intervals, with statistical differences defined as *P* < 0.05.

## Results

Eighty-six patients were included in this study, and the demographic characteristics and segmental distribution of injuries of all patients are shown in Table 1. The mean age of all patients was 46.8 ± 9.5 years, including 55 male patients (64.0%), 31 female patients (36.0%), and the male to female ratio was 1.77:1. The injury was due to falls from height in 44 patients (51.2%) and other causes such as serious crush injuries and road traffic accidents in 42 patients (48.8%). There were 16 patients (18.6%) with burst fractures of T11 or T12 and 70 patients (81.4%) with burst fractures of L1 or L2. The preoperative ASIA scale was E in all patients, the preoperative TLICS was greater than or equal to four in all patients, and the preoperative LSC was less than seven. All patients were operated on within a week. The patients were followed up for an average of 17.8 months. Radiographic examination at the last follow-up visits or before removal of the internal fixation in all patients showed that the fractures reached bony union and had no broken nails. According to kyphotic Cobb angle change, 47 out of 86 patients were included in the no loss of correction group, while the remaining 39 were included in the loss of correction group. The univariate analysis of the variables in the table was performed in Table 2. Univariate analysis showed no statistically significant differences in age, gender, cause of injury, injured segment, or preoperative VWA between the two groups (*P* > 0.05). Finally, three variables, including the presence or absence of vertical laminar fracture (*P* < 0.05), preoperative AVH (*P* < 0.05), and preoperative Cobb angle (*P* < 0.05), were selected to proceed to the next binary logistic regression analysis. By binary logistic regression analysis, presented in Table 3, we found that the presence or absence of vertical laminar fracture (OR: 7.74; 95% CI: 2.41-24.86; *P* = 0.01) and preoperative Cobb (OR: 1.13; 95% CI: 1.01-1.26; *P* = 0.033) were independent risk factors for postoperative loss of correction in thoracolumbar burst fractures. Preoperative AVH% (OR: 1.00; 95% CI: 0.95-1.06; *P* = 0.954) was not an independent risk factor for postoperative loss of correction.

**Table 1:** Demographic data

Variables		
Number of participants, *n*	86	
Gender,[*n* (%)]		
Male	55	(64.0%)
Female	31	(36.0%)
Aetiology,[*n* (%)]		
Fall from height	44	(51.2%)
Other	42	(48.8%)
Location,[*n* (%)]		
T11	2	(2.3%)
T12	14	(16.3%)
L1	48	(55.8%)
L2	22	(25.6%)
Age/years, x ± s	46. 8 ± 9.5	
Follow up time/months, x ± s	17.8 ± 4.5

**Table 2:** Results of univariate analysis of clinical and radiological data between groups

Variables	A group (recurrent kyphosis) 39	B group (Non-recurrent kyphosis) 47	*P* Value
Gender, *n*			0.635
Male	26	29	
Female	13	18	
Location, *n*			0.487
T11/T12	6	10	
L1/L2	33	37	
Aetiology, *n*			0.984
Fall from height	20	24	
Other	19	23	
Laminar fractures, *n*			*
With laminar fractures	33	20	
Without laminar fractures	6	27	
Age/years, x ± s	48.7 ± 9.3	45.2 ± 9.5	0.086
Follow up time/months, x ± s	18.1 ± 5.2	17.5 ± 3.9	0.540
Pre-AVH%, x ± s	59.9 ± 10.4	66.9 ± 12.9	0.007
Pre-cobb angle/°,x ± s	16.3 ± 5.5	12.8 ± 5.2	0.003
Pre-VWA/°,x ± s	17.0 ± 5.9	15.1 ± 5.1	0.115

*:*P*<0.01

**Table 3:** Logistic regression analysis of risk factor for recurrent kyphosis

Variables	*P* Value	OR	95%CI
Laminar fractures	0.001	7.74	2.41-24.86
Pre-AVH%	0.954	1.00	0.95-1.06
Pre-cobb angle/°	0.033	1.13	1.01-1.26

To determine whether the morphology of the vertical laminar fracture influenced the loss of correction after thoracolumbar burst fractures with associated laminar fractures, we screened 53 patients with associated laminar fractures. According to previous criteria, we divided them into a no loss group and a loss group, 20 of whom were included in the no loss group and the remaining 33 in the loss group. As shown in Table 4, statistical analysis using the variables in the table showed that there was no statistical difference in age, gender, etiology, injured segment, pre-AVH%, pre-cobb angle, pre-VWA, injury degree of laminar fractures in the horizontal plane between the two groups (*P* > 0.05). There was a statistically significant difference in the pattern of laminar fractures between the two groups (*P* < 0.05), suggesting a statistical difference between the effect of full-length and partial-length laminar fractures on the loss of correction after thoracolumbar burst fractures with laminar fractures.

**Table 4:** Results of univariate analysis of clinical and radiological data between groups

Variables	A group (recurrent kyphosis) 33	B group (Non-recurrent kyphosis) 20	*P* Value
Gender, *n*			0.284
Male	23	11	
Female	10	9	
Location, *n*			0.097
T11/T12	5	7	
L1/L2	28	13	
Aetiology, *n*			0.863
Fall from height	19	12	
Other	14	8	
Injury degree of laminar fractures in the the coronal plane, *n*			0.035
Full-length laminar fractures	23	8	
Part-length laminar fractures	10	12	
Injury degree of laminar fractures in the the axial plane, *n*			0.807
Complete laminar fractures	17	11	
Incomplete laminar fractures	16	9	
Age/years, x ± s	49.1 ± 9.6	43.4 ± 10.8	0.052
Follow up time/months, x ± s	17.6 ± 4.5	18.0 ± 2.9	0.693
Pre-AVH%, x ± s	59.0 ± 10.7	60.9 ± 12.9	0.587
Pre-cobb angle/°,x ± s	16.4 ± 5.5	15.8 ± 5.9	0.708
Pre-VWA/°,x ± s	16.9 ± 6.0	16.5 ± 5.9	0.839

## Discussion

The thoracolumbar burst fracture is characterized by a falling height on the anterior margin of the vertebral body and damage to the posterior margin of the vertebral body. Upon encroachment of the posterior vertebral wall and posterior ligamentous complex, the posteriorly displaced fracture fragment encroaches into the spinal canal [18, 19] with a high risk of neurological impairment [20]. Mohamed M. Aly et al. [21] showed that the absence of horizontal laminar fracture, spinous process fracture, interspinous widening > 4 mm, and facet joint malignment has a high negative predictive value of PLC injury. When the interpedicular distance between the two sides of the vertebral body widens, laminar fractures result when the force exceeds the durability of the lamina. Tisot et al. [22] analyzed 92 thoracolumbar burst fracture patients with associated laminar fractures and found that injury severity and spinal canal encroachment were significantly higher (*P* < 0.001). These findings confirm that laminar fractures can measure the severity of fractures. A growing body of evidence suggests that thoracolumbar burst fractures associated with laminar fractures have a significantly increased risk of dural tears and impaired neurological functioning [23–26]. Vertebral bodies are subject to different axial forces transmitted to the lamina across the pedicle, producing laminar fractures with different laminar fractures under different forces. The laminar fracture was a reliable factor to assess the severity of spinal fracture.

In this study,patients with full-length laminar thoracolumbar burst fractures were at greater risk of postoperative loss of correction than those with partial-length laminar fractures (*P* < 0.05). As the longitudinal extrusion at the time of the fracture increases, so does the force applied to the lamina. In laminar fractures that present with full-length and partial-length laminar fractures at different force magnitudes, full-length laminar fractures tend to cause greater injury than part-length laminar fractures. There is a marked difference between the impact of full-length and partial-length laminar fractures on the postoperative correction. Statistical analyses of patients with associated vertical laminar fracture showed that Cobb was no longer an independent predictor of correction loss. This could be attributed to the difference in the samples for the two statistics. The first sample consisted of 85 patients with thoracolumbar burst fractures, and the second sample consisted of 53 patients with vertical laminar fracture. Considering that patients with vertical laminar fracture typically have higher degrees of fracture injury, and Cobb is already small at this point, the difference in Cobb between those with and without vertical laminar fracture must be smaller. This explains the variance in preoperative Cobb’s scores between the two statistical analyses. Our study may be subject to statistical error due to insufficient sample size and homogeneity. In our subsequent studies, we attempted to collect data from multiple sites and conducted prospective studies to minimize error.

Anterior column injury to the vertebral body is a important cause of loss of correction following thoracolumbar burst fracture surgery. The instability of the anterior column of the spine could be attributed primarily to disc injuries and intravertebral ‘Eggshell’ defects. Disc and posterior ligament complex injuries are frequently associated with severe thoracolumbar burst fractures. Wang et al. [27] suggested that the loss of postoperative correction is associated with reduced intervertebral space due to disc collapse above the injured vertebra. The patients in this study had mild to moderate fractures with an LSC score of less than 7. Therefore, disc injury was less likely, and all patients had a preoperative MRI to exclude disc injury, so this factor was excluded from this study. The posterior ligament complex is essential for maintaining spine stability [28, 29]. A recent study has shown that the analysis of combined CT findings could improve the ability to confirm or rule out PLC injury [30]. Previous studies have been based on the laminar fractures observed in the horizontal plane on CT. In contrast, the laminopathic morphology presented by multiple planes on CT and the relationship of this morphology to the loss of postoperative correction has never been investigated. We observed and described multiple laminar fractures in 2D CT reconstructions, dividing them into four categories based on the multiple planes present. Our study confirmed that the lamina Bifida and the preoperative Cobb were risk factors for loss of correction after thoracolumbar burst fracture surgery.

The objective of this study was to determine whether concomitant vertical laminar fracture are associated with loss of correction after thoracolumbar burst fracture surgery. Previous studies have well documented that the distance between the injured pedicles is an independent risk factor for internal fixation failure [31]. There is also evidence that preoperative Cobb is a risk factor for loss of postoperative correction. Preoperative Cobb’s hours are frequently associated with severe compression of the vertebral body, the interior of which is composed of cancellous bone. While intraoperative fluoroscopy suggests that the vertebral height can be restored when pedicle screws indirectly reduce the fracture, the cancellous bone inside the vertebral body cannot be fully restored.

There are several limitations to this study. The study included only patients from one hospital’s spine surgery department and a large sample multicenter study remains required to reduce the error. The second reason is that this study was retrospective, and the probable loss of clinical data might lead to the appearance of an error because of the insufficient sample size. Third, since patients with thoracolumbar burst fractures complained of extreme pain and could not stand, they had to take spinal radiographs lying down. As patients’ clinical symptoms improved following surgery, standing was often the preferred position to take spinal radiographs. In this study, the radiograph position was not controlled, which may have affected the accuracy of the results. Fourth, the follow-up time after implant removal was short. That we had only 2 reviewers is another potential limitation. While we conducted a consensus reading to achieve a high degree of concordance, we acknowledge that this reading might be less reproducible in reviewers with less experience and in reviewers with no training.

## Conclusions

1. This study was conducted to determine whether or not simultaneous lamina splitting and preoperative Cobb are independent risk factors for loss of postoperative correction in patients with thoracolumbar burst fractures.
2. There was a statistical difference between full-length and partial-length laminar fractures on the loss of postoperative correction of thoracolumbar burst fractures with associated vertical laminar fracture.
3. There was no statistical difference in the effect of vertical laminar fracture morphology at the horizontal plane on the loss of postoperative correction of thoracolumbar burst fractures with associated vertical laminar fracture.
4. This study did not associate patient age, gender, cause of injury, injured segment, preoperative AVH%, and preoperative VWA with postoperative loss of Cobb correction.

## Data Availability

The datasets generated during and analyzed during the current study are not publicly available due to the protection of patient privacy but are available from the corresponding author on reasonable request.
